# A Novel Super-Junction DT-MOS with Floating p Regions to Improve Short-Circuit Ruggedness

**DOI:** 10.3390/mi14101962

**Published:** 2023-10-21

**Authors:** Sujie Yin, Wei Cao, Xiarong Hu, Xinglai Ge, Dong Liu

**Affiliations:** 1 School of Electrical Engineering, Southwest Jiaotong University, Chengdu 611756, China; yinsujie@my.swjtu.edu.cn (S.Y.); caowei123@my.swjtu.edu.cn (W.C.); xlge@home.swjtu.edu.cn (X.G.); 2 School of Science, Xihua University, Chengdu 610039, China; hxr2013@mail.xhu.edu.cn

**Keywords:** 4H-SiC, double-trench SiC MOSFET, short-circuit (SC), super-junction (SJ)

## Abstract

A novel super-junction (SJ) double-trench metal oxide semiconductor field effect transistor (DT-MOS) is proposed and studied using Synopsys Sentaurus TCAD in this article. The simulation results show that the proposed MOSFET has good static performance and a longer short-circuit withstand time (*t*_sc_). The super-junction structure enables the device to possess an excellent compromise of breakdown voltage (*BV*) and specific on-resistance (*R*_on,sp_). Under short-circuit conditions, the depletion of p-pillar, p-shield, and floating p regions can effectively reduce saturation current and improve short-circuit capability. The proposed device has minimum gate-drain charge (*Q*_gd_) and gate-drain capacitance (*C*_gd_) compared with other devices. Moreover, the formation of floating p regions will not lead to an increase in process complexity. Therefore, the proposed MOSFET can maintain good dynamic and static performance and short-circuit ability together without increasing the difficulty of the process.

## 1. Introduction

It is known that silicon carbide material has superior critical breakdown electric field strength, higher thermal conductivity, higher saturated electron drift velocity, and a wider band gap compared with silicon [1,2,3]. Therefore, compared with silicon devices, silicon carbide devices can better compromise breakdown voltage and specific on-resistance to achieve better performance [4]. SiC MOSFET is considered to be a promising competitor to Si IGBT, but it is accompanied by reliability problems under strict service conditions [5,6,7]. The short-circuit withstand time of SiC MOSFET is shorter than Si IGBT owing to smaller die size and higher saturation current [8,9,10]. Short-circuit performance is an important reliability problem of SiC devices. At the moment of short-circuit, the device needs to withstand high voltage and current. If the short-circuit capability of the device is inadequate, the performance of the device will be degraded, or even burned [11]. Therefore, the short-circuit reliability of devices has widely been a concern of researchers.

The trench MOSFET has higher power density than planner MOSFET, but it has the disadvantage of a high electric field at the bottom oxide [12]. The design of the double-trench MOSFET (DT-MOS) can better protect the gate oxide and reduce the oxide electric field [13]. Because of the higher power density of the DT-MOS, the short-circuit robustness is relatively poor. However, there are few studies on short-circuit reliability of DT-MOS at present. In paper [14], the DPCSL-MOS is proposed to reduce the saturation current of the device and improve SC ruggedness. The SWITCH-MOS improves short-circuit capability to be consistent with the SiC trench MOSFET via a high Schottky barrier (1.75 eV) in paper [15]. The SiC MOSFET with embedded auto-adjust JFET is proposed to improve device short-circuit capability through the reduction in the saturation current [16]. In addition, deep p-well [17] is also used to enhance short-circuit capability. However, further optimization is needed to improve the short-circuit capability of the device.

In this paper, a super-junction DT-MOS with floating p regions is proposed and simulated with Synopsys Sentaurus TCAD [18]. According to the simulation results, the proposed structure improves the gate oxide electric field, effectively reduces the saturation current, and enhances short-circuit reliability. In addition, the proposed structure has lower switching losses due to lower *Q*_gd_ and *C*_gd_. The effects of doping concentration (*N*_p-region_), depth (*h*), and width (*w*) in the floating p regions are studied on the specific on-resistance (*R*_on,sp_), breakdown voltage (*BV*), and saturation current density (*I*_sat_). The relationship between static performance and short-circuit ruggedness is considered as a compromise to achieve the optimal device structure design.

## 2. Device Structure

**Figure 1:**
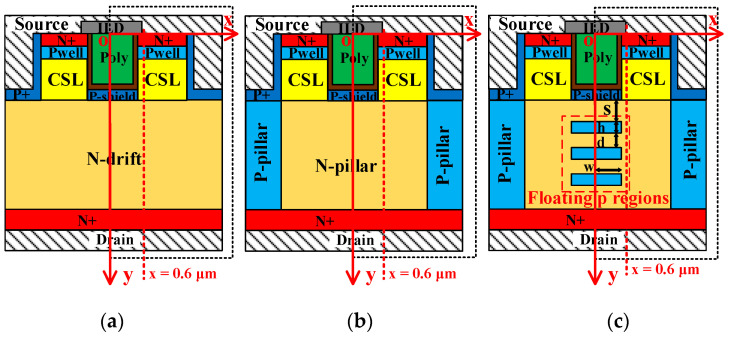
Cross-sectional structures of (**a**) DT-MOS, (**b**) SJ-DTMOS, and (**c**) proposed MOSFET (drawing not to scale).

Figure 1 shows the cross sections of double-trench MOSFET (DT-MOS), super-junction double-trench MOSFET (SJ-DTMOS), and proposed MOSFET. The N-drift and N-pillar thicknesses are 10 μm, in which the N-drift doping concentration of DT-MOS is 8 × 10^15^ cm^−3^, and the N-pillar doping concentrations of SJ-DTMOS and proposed MOSFET are 6 × 10^16^ cm^−3^ and 5 × 10^16^ cm^−3^, respectively. For three structures, the channel length of 0.5 μm, p-well doping concentration of 2 × 10^17^ cm^−3^, and oxide thickness of 50 nm are adopted. The P-shiled layer is used to reduce the gate oxide electric field and enhance the reliability of gate oxide. The depth and doping concentration of the P-shield layer are 0.3 μm and 2 × 10^18^ cm^−3^, respectively. The specific on-resistance increases due to the JFET effect caused by the P-shield layer. Therefore, the current spreading layers (CSLs) with doping concentrations of 4 × 10^16^ cm^−3^ are introduced to improve the conduction capability of the device. The device structure parameters are summarized in Table 1.

Considering the accuracy of the simulation results, many physic models containing the avalanche model (Okuto), anisotropy model, incomplete ionization model, Fermi model, high field velocity saturation model, Auger recombination, and Shockley–Read–Hall (SRH) recombination are adopted in the simulation [19]. The anisotropy model takes into account the differences of material properties in different directions and can better describe the anisotropy of 4H-SiC. When the impurity energy level is deeper than the thermal energy, the impurity may not be completely ionized, so it is necessary to consider the incomplete ionization model. The Fermi model uses Fermi–Dirac statistics. The high field velocity saturation model is used to describe the carrier drift velocity of a strong electric field. The recombination at high carrier concentration is considered in Auger recombination, and Shockley–Read–Hall recombination is used to describe the recombination through deep defect levels in the gap. They are all used in the mobility model. The mobility model also adopts the Inversion and Accumulation Layer Mobility Model (IALMob) including the effects of Coulomb impurity scattering, phonon scattering, and surface roughness scattering. Moreover, the thermodynamic model is also considered in short-circuit simulations to describe the electrothermal coupling behavior.

## 3. Simulation Results and Discussion

**Figure 2:**
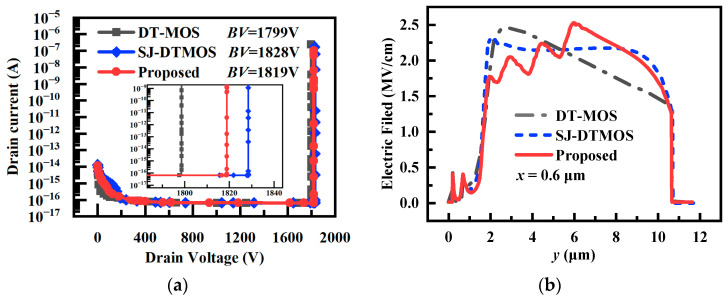

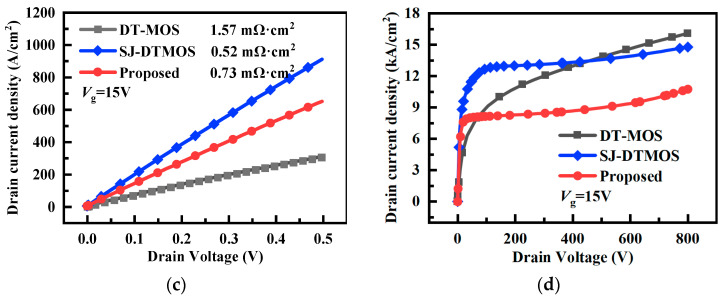
The simulation results of (**a**) *BV* characteristics, (**b**) vertical electric field distribution along *x* = 0.6 μm at breakdown, (**c**) *I*-*V* characteristics at low drain voltages, and (**d**) *I*-*V* characteristics (*V*_ds_ from 0 to 800 V).

Figure 2a exhibits the breakdown characteristics. The distribution of the electric field is shown in Figure 2b. It can be seen that the vertical electric field distribution of SJ-DTMOS is uniform due to the complete depletion of the N-pillar and P-pillar. Therefore, even when the doping concentration of the N-pillar is greater than the concentration of the N-drift of DT-MOS, the breakdown voltage of the super-junction MOSFET is slightly higher than DT-MOS. The breakdown voltage of the proposed MOSFET slightly decreases due to the charge imbalance caused by the introduction of the floating p regions. There are three jags in the vertical electric field curve of the proposed device in Figure 2b, which is caused by the introduction of floating p regions. When the device is in a high-voltage state, the floating p regions and n-pillar deplete each other to form a lateral electric field. The electric field near the floating p regions is equal to the combination of the vertical electric field and the lateral electric field. Therefore, passing through the vicinity of three floating p regions along the vertical direction, the electric field increases and decreases three times.

The I-V characteristics at different voltage levels are given in Figure 2c,d. The specific on-resistances (*R*_on,sp_) of three devices are extracted at *I*_d_ = 100 A/cm^2^, and summarized in Figure 2c. The depletion of the introduced floating p regions leads to the narrowing of the current path and the increase in the *R*_on,sp_. However, due to the advantages of the super-junction structure, its *R*_on,sp_ is still far less than DT-MOS. In the high-voltage state, the expansion of the depletion layer in the floating p regions can effectively reduce the saturation current density (*I*_sat_), so the proposed MOSFET has the minimum *I*_sat_ compared with DT-MOS and SJ-DTMOS.

**Figure 3:**
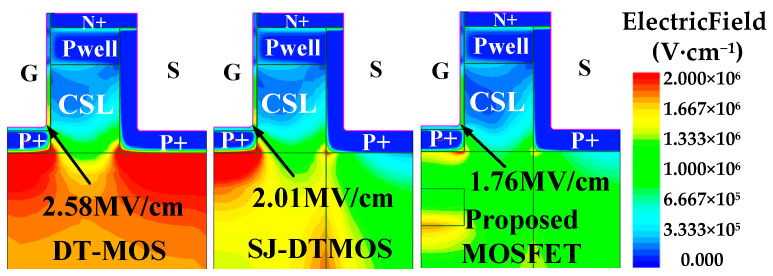
Electric field contours of the three devices with *V*_d_ = 1200 V.

Figure 3 illustrates the electric field contours of three devices under *V*_d_ = 1200 V. The maximum electric fields of oxide (*E*_ox-m_) are 2.04 MV/cm, 2.01 MV/cm and 1.76 MV/cm, respectively. The proposed MOSFET has minimum *E*_ox-m_ due to the introduction of floating p regions causing changes in the distribution of the electric field inside the device. The p+ shield at the bottom of gate oxide reduces the *E*_ox-m_ to a safe area (<3 MV/cm) [20] and ensures the long-term reliability of the devices.

**Figure 4:**
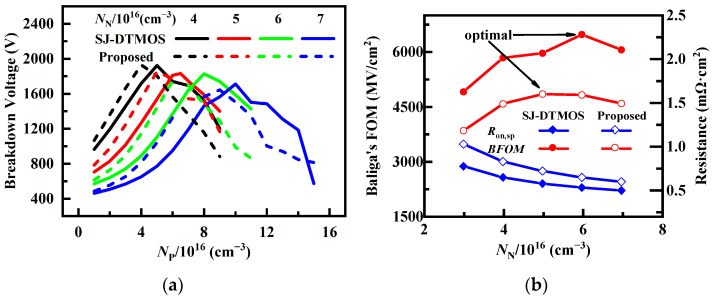
(**a**) Effect of the *N*_P_ on *BV* of SJ-DTMOS and the proposed MOSFET with a different *N*_N_. (**b**) Relationship between the *BFOM* and *R*_on,sp_ of two devices under different *N*_N_.

The doping concentrations of the P-pillar and N-pillar (*N*_P_ and *N*_N_) in the SJ structure will affect the static performance of the device. It is important to choose the appropriate *N*_P_ and *N*_N_ to achieve the best performance. The effects of the *N*_P_ and *N*_N_ on *BV* are illustrated in Figure 4a. It can be seen that the breakdown voltage (*BV*) first increases and then decreases with the increase in *N*_P_, and there is an optimal *N*_P_ to maximize *BV*. Moreover, *BV* decreases with increasing *N*_N_ owing to the increase in the lateral electric field [21]. Figure 4b shows the effect of *N*_N_ on Baliga’s figures of merit (*BFOM*) and specific on-resistance (*R*_on,sp_). Due to the introduction of floating p regions, the proposed MOSFET has a slightly larger *R*_on,sp_ than SJ-DTMOS at the same *N*_N_. Obviously, the optimal *N*_N_ of SJ-DTMOS and the proposed MOSFET are, respectively, 6 × 10^16^ cm^−3^ and 5 × 10^16^ cm^−3^, which can largely compromise *BV* and *R*_on,sp_.

**Figure 5:**
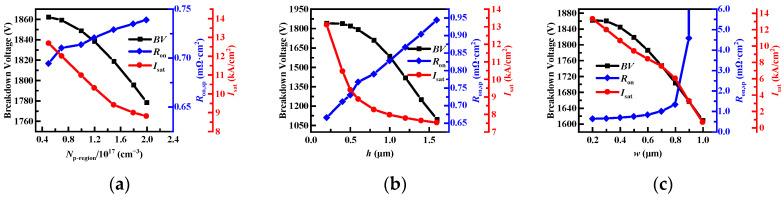
The effects of (**a**) *N*_p-region_, (**b**) *h*, and (**c**) *w* on the static characteristics of the proposed MOSFET.

The influence of the doping concentration of floating p regions (*N*_p-region_) for the *BV*, *R*_on,sp_, and *I*_sat_ of the proposed MOSFET is discussed. From Figure 5a, it can be seen that *BV* becomes smaller monotonously with increasing *N*_p-region_ due to an aggravated charge imbalance caused by floating p regions. In addition, the increased *N*_p-region_ results in a monotonous increase in *R*_on,sp_ and a monotonous decrease in saturation current density (*I*_sat_), because the introduced floating p regions cause the current path-narrowing. For the proposed MOSFET, the *N*_p-region_ is chosen to 1.5 × 10^17^ cm^−3^ for achieving a good tradeoff between static performance and *I*_sat_. Considering the influence of process deviation, the effects of the floating p region’s width (*w*) and depth (*h*) on the performance of the proposed device are shown in Figure 5b,c. As expected, the increases in *w* and *h* cause the increase in *R*_on,sp_ and the decrease in saturation current density *I*_sat_. In addition, it is worth noting that *w* greater than 0.8 μm could result in a rapid increase in *R*_on,sp_, or even make it unable to turn on. With the increase in *w* and *h*, it will lead to different degrees of decrease in *BV*. Therefore, *w* and *h* should be controlled within a reasonable range to achieve a better compromise between *BV*, *R*_on,sp_, and *I*_sat_.

**Figure 6:**
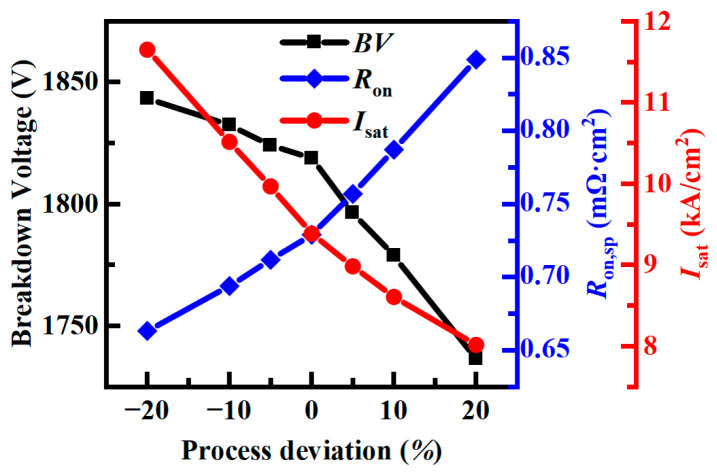
The effect of process deviation on the breakdown voltage (*BV*), specific on-resistance (*R*_on,sp_), and saturation current density (*I*_sat_) of the device.

Considering the process deviation of ion implantation, the effects of different floating p regions’ width (*w*) and depth (*h*) on device performance are simulated. Figure 6 shows the static characteristics of the proposed device under different process deviations with the floating p region’s width *w* = 0.5 μm and depth *h* = 0.5 μm as the basis when the process deviation is 10%, *w* = 0.55, and *h* = 0.55. It can be seen that the breakdown voltage of the device decreases with the process deviation from −20% to +20%. This is because the area of the floating p regions becomes larger, which aggravates the charge imbalance. When the process deviates from −20% to +20%, the floating p region’s area becomes larger and the current path becomes narrow, so the specific on-resistance will increase, while the saturation current density will decrease. When the deviation is between −20% and +20%, the breakdown voltage will not drop sharply, and the specific on-resistance will not rise sharply, so it has a wider process window. Good static characteristics and short-circuit capacity can be obtained by adjusting the process.

**Figure 7:**
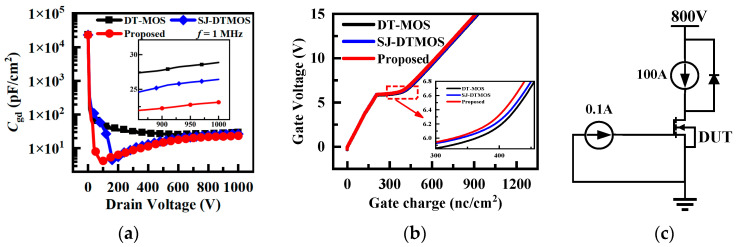
(**a**) Capacitance characteristics for the three studied devices. (**b**) Gate charge characteristic curves of three studied devices. (**c**) Gate charge test circuit.

Figure 7 shows the capacitance and gate charge characteristics of the three studied devices. Gate-drain capacitance (*C*_gd_) plays a key role in the switching speed and losses of the device. The proposed structure has minimal *C*_gd_ (23.1 pF/cm^2^). The *C*_gd_ of the SJ-DTMOS and the proposed MOSFET show a sharp drop and then rise, and the phenomenon in SJ devices is discussed in the literature [22,23]. But, it is worth noting that the introduction of floating p regions allows the *C*_gd_ to drop more rapidly at low *V*_ds_. Therefore, the proposed MOSFET possesses the lowest gate-drain charge (*Q*_gd_, *Q*_gd_ = ∫ *C*_gd_ d*V*_ds_). The detailed parameters of three devices are summarized in Table 2.

**Figure 8:**
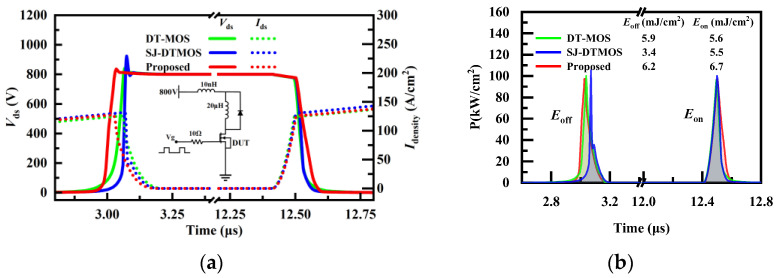
(**a**) Voltage and current waveforms of the three studied MOSFETs during double-pulse testing. (**b**) Power losses of the three studied MOSFETs during turn-on and turn-off.

Considering the influence of the introduction of floating p regions on the dynamic performance of the proposed device, the double-pulse test simulation of the three studied devices is carried out. Figure 8a shows the voltage and current waveforms of the three studied devices. The double-pulse test circuit is shown in Figure 8a, in which the gate resistance is 10 Ω, the load inductance is 20 μH, the loop stray inductance is 10 nH, and the gate pulse voltage is 15/0 V. The small-signal capacitance and dynamic capacitance of the super-junction MOSFET are different [23]. Even though the proposed structure has the smallest *C*_gd_ in the small-signal measurement, the introduction of floating p regions may result in a larger dynamic *C*_gd_, so the proposed structure has a longer turn-on and -off time. It can be seen that the proposed structure has a greater turn-off loss owing to the longer time of the proposed MOSFET during the period of high voltage and high current. Similarly, due to the introduction of floating p regions, resulting in a smaller voltage change rate dV/dt during the turn-on period, the turn-on loss of the proposed MOSFET is slightly larger than that of the other two devices. The data related to the dynamic characteristics of the three studied devices are recorded in Table 2.

**Figure 9:**
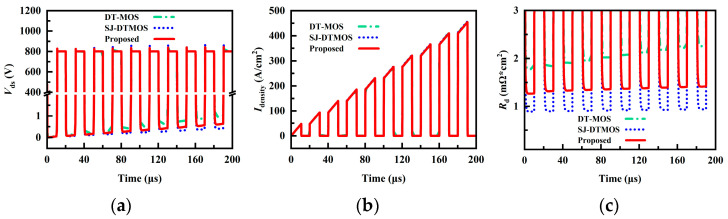
Multiple switching of the three studied MOSFETs: (**a**) *V*_ds_, (**b**) *I*_density_, (**c**) *R*_d_.

Figure 9 shows the voltage (*V*_ds_), current density (*I*_density_), and dynamic-specific on-resistance (*R*_d_) waveforms of the three studied devices under multiple switching. The three structures have similar current density waveforms. The forward voltage drop increases with the increase in the load current density, which is consistent with the I-V characteristic curve in Figure 2c. The waveform of *R*_d_ during the switching operation is shown in Figure 9c. It can be seen that the *R*_d_ of DT-MOS and the proposed MOSFET are degraded during the switching process. The *R*_d_ of the three studied devices at the first and tenth turn-on is summarized in Table 2.

**Figure 10:**
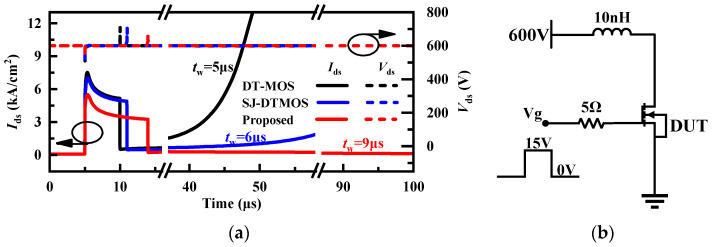
(**a**) Voltage and current waveforms of the three MOSFETs during the short circuit. (**b**) Short-circuit test circuit.

The short-circuit (SC) test results of the DT-MOS, SJ-DTMOS, and proposed MOSFET are shown in Figure 10a. Figure 10b shows the short-circuit test circuit used in the simulation, where the gate resistance and stray inductance are 5 Ω and 10 nH, respectively [21]. The gate pulse time of the three devices is 5 μs, 6 μs, and 9 μs, respectively. The short-circuit withstand time (*t*_sc_) of each device is determined by applying a single pulse of 15 V/0 V to the gate contact for the time until the device fails. The proposed MOSFET has the longest *t*_sc_ and the lowest saturation current density due to the narrowing current path caused by the depletion layer expansion of floating p regions during short-circuit. According to Figure 10a, when the short-circuit time exceeds the *t*_sc_ of the device, the device current will not drop to 0 with time, and the device will fail due to thermal runaway caused by high temperatures (over 1800 K).

**Figure 11:**
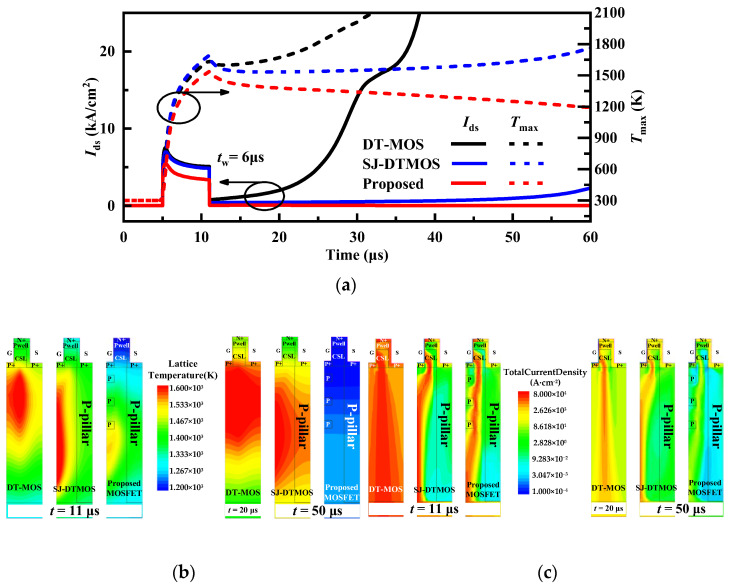
(**a**) Total current and temperature waveforms for the three studied devices during short-circuit conditions (600 V dc bus voltage). (**b**) Lattice temperature distribution of the DT-MOS (at *t* = 11 μs, 20 μs), the SJ-DTMOS (at *t* = 11 μs, 50 μs), and the proposed MOSFET (at *t* = 11 μs, 50 μs). (**c**) Total current density distribution of the DT-MOS (at *t* = 11 μs, 20 μs), the SJ-DTMOS (at *t* = 11 μs, 50 μs), and the proposed MOSFET (at *t* = 11 μs, 50 μs).

The introduced thermodynamic model includes the calculation of lattice temperature, taking into account self-heating, and the change in lattice temperature leads to the change in carrier concentration and mobility, which affects the performance of the device. After the MOSET short-circuit (SC), the short-circuit current increases rapidly, and then the short-circuit current decreases caused by the decrease in carrier mobility due to the increase in the lattice temperature [24]. The carrier density climbs with the higher lattice temperature, resulting in many holes, so that it can not be turned off completely, and there is a tail current. The trailing current leads to the generation of more heat and forms the positive feedback of electrothermal. When the SC current exceeds the threshold, it will trigger the conduction of parasitic transistors, further aggravate the positive feedback and increase lattice temperature, and finally lead to thermal runaway, as shown in DT-MOS in Figure 11a.

Figure 11a shows the current density and temperature waveforms of the short circuit (short-circuit pulse width *t*_w_ = 6 μs). The temperature distribution and current density distribution at the end of the short circuit (*t* = 11 μs) and after a while (*t* = 20 μs or 50 μs) are shown in Figure 11b,c. It can be seen that, due to the depletion layer expansion of the floating p-region at high voltage, the short-circuit current of the proposed structure is obviously smaller than the other devices. It can be seen that the turn-off is not complete, and there is a tail current in the device. The power losses caused by the tail current caused the lattice temperature of the device to rise, eventually leading to thermal runaway. This happened to both DT-MOS and SJ-DTMOS in Figure 11a. Attributed to the smaller current, the lattice temperature of the proposed MOSFET is significantly lower than that of other devices at all moments, which effectively suppresses the electrothermal coupling effect and improves the short-circuit ability of the proposed device.

Table 2 exhibits the performance comparison of the three devices. It can be seen that the proposed MOSFET has the lowest *Q*_gd_ and *C*_gd_. However, it can be seen through double-pulse test simulation that the switching loss of the proposed structure is the largest due to the smaller voltage and current change rates. The proposed MOSFET has the longest *t*_sc_ and good static performance. Therefore, slightly larger switching losses can be accepted due to the excellent short-circuit capability.

**Figure 12:**
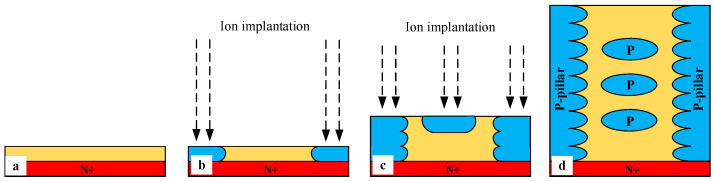
The key processing steps to fabricate the SJ structure with floating p regions. (**a**) Substrate wafer with first epitaxy. (**b**) Masked aluminum (Al) implantation to form p-pillar. (**c**) Masked Al implantation to form p-pillar and floating p regions. (**d**) Multiple epitaxy and ion implantation to form SJ structure with floating p regions.

Since the SJ structure is formed of multiple epitaxy layer growth and ion implantation [11,25,26], the SJ process is compatible with the floating p region’s process. Figure 12 shows the formation of the SJ structure and floating p regions using multilayer epitaxy and ion implantation techniques. It can be seen that floating p regions can be obtained only by increasing the ion implantation window. Therefore, the process of the proposed structure is almost the same as the SJ process, which will not increase the process difficulty and is realizable.

**Figure 13:**
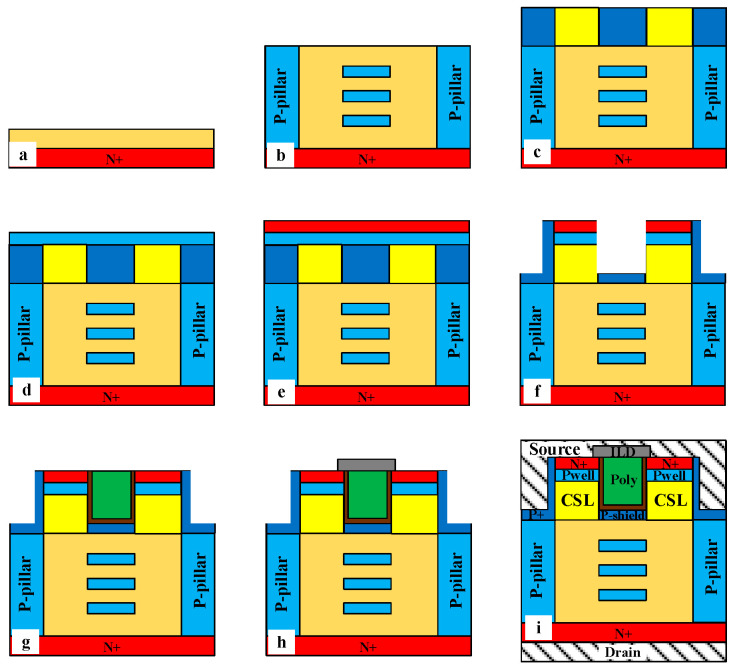
The fabrication process flow of the proposed MOSFET: (**a**) Substrate wafer with first epitaxy. (**b**) Super-junction structure with floating p regions formed through multiple epitaxy and ion implantation. (**c**) Epitaxy and ion implantation form CSL and p+ region. (**d**) Formation of p-well through epitaxy. (**e**) Formation of N+ region through epitaxy. (**f**) Trench etching. (**g**) Thermal oxidation of gate oxide and deposition of polysilicon gate. (**h**) Deposition of the interlayer dielectric (ILD). (**i**) Metallization forms contacts.

A simple and feasible fabrication process flow of the proposed MOSFET is provided in Figure 13. First, the fabrication process starts with the substrate wafer with the first epitaxy in Figure 13a. Then, the super-junction structure with floating p regions in Figure 13b is formed through multiple epitaxy and ion implantation as shown in Figure 12. Figure 13c shows that the current spreading layers (CSLs) and p+ regions are formed through epitaxy and ion implantation. Figure 13d,e shows that p-well regions and n+ regions are formed through epitaxy. The thermal oxidation to form gate oxide and the deposition to form polysilicon are shown in Figure 13g. The interlayer dielectric (ILD) is formed through deposition in Figure 13h. Finally, contacts are formed through the metallization process in Figure 13i.

## 4. Conclusions

In this paper, a novel super-junction (SJ) double-trench SiC MOSFET with floating p regions is proposed and studied through TCAD simulation. The simulation results show that the proposed structure has higher gate oxide reliability due to the minimum oxide electric field caused by the introduction of the floating p region changing the electric field distribution inside the device. In this paper, the effects of the floating p regions’ width, depth, and concentration on the performance of the device are studied, and the optimal value is determined, so that the proposed device has excellent static characteristics and short-circuit ability. In addition, considering the influence of process deviation, the proposed MOSFET under different process deviations is simulated. The simulation results show that the SJ-DTMOS with floating p regions has a large process window, and the performance of the device is only slightly changed under the process deviation of −20% to 20%, and a good compromise between static characteristics and short-circuit ability can be achieved.

In addition to the static characteristics, the gate-drain capacitance (*C*_gd_) and gate-drain charge (*Q_g_*_d_) of the three studied devices are compared, and the proposed MOSFET has the smallest *C*_gd_ and *Q_g_*_d_. Unfortunately, due to the smaller current changing rate and voltage changing rate, the proposed structure possesses larger switching losses than the other two structures. However, the simulation results show that the short-circuit withstand time *t*_sc_ of the proposed MOSFET increases by 125% and 80% compared with DT-MOS and SJ-DTMOS, respectively. Therefore, the switching losses of the proposed MOSFET are acceptable due to its excellent short-circuit ability and higher gate oxide reliability. The fabrication processes of the SJ structure and floating p regions are compatible, which can be realized through multiple epitaxy and multiple ion implantation. A simple and feasible process of super-junction DTMOS with floating p regions is given in this paper.

## Figures and Tables

**Table 1:** The structure parameters of the three devices.

Device Parameters	DT-MOS	SJ-DTMOS	Proposed
N-pillar or N-drift doping (cm^−3^)	8 × 10^15^	6 × 10^16^	5 × 10^16^
P-pillar doping (cm^−3^)	—	8 × 10^16^	5 × 10^16^
Number of floating p-region (*n*)	—	—	3
Floating p-region doping (cm^−3^)	—	—	1.5 × 10^17^
*s* (μm)	—	—	0.5
*h* (μm)	—	—	0.5
*w* (μm)	—	—	0.5
*d* (μm)	—	—	1.0

**Table 2:** Device performance comparison.

Symbol	DT-MOS	SJ-DTMOS	Proposed MOSFET
*BV* (V)	1799	1829	1819
*R*_on,sp_ ^1^ (mΩ·cm^2^)	1.57	0.52	0.73
*E*_ox_ ^2^ (MV/cm)	2.04	2.01	1.76
*Q*_gd_ (nC/cm^2^)	179	153	144
*C*_gd_ ^3^ (pF/cm^2^)	28.8	26.4	23.1
*E_on_* ^4^ (mJ/cm^2^)	5.6	5.5	6.7
*E_off_* ^5^ (mJ/cm^2^)	5.9	3.4	6.2
*E_total_* ^6^ (mJ/cm^2^)	11.5	8.9	12.9
*R*_d1_ ^7^ (mΩ·cm^2^)	1.78	0.89	1.27
*R*_d10_ ^8^ (mΩ·cm^2^)	2.26	0.93	1.41
*t*_sc_ (μs)	4	5	9

^1^ *R*_on,sp_ is calculated at *I*_ds_ = 100 A/cm^2^. ^2^ *E*_ox-m_ is extracted at *V*_d_ = 1200 V. ^3^ *C*_gd_ is extracted at *V*_d_ = 1000 V. ^4^ *E_on_* is the power loss of turn-on. ^5^ *E_off_* is the power loss of turn-off. ^6^ *E_total_* is the sum of *E_on_* and *E_off_*. ^7^ *R*_d1_ is the dynamic-specific on-resistance at the first turn-on. ^8^ *R*_d10_ is the dynamic-specific on-resistance at the 10th turn-on.

## Data Availability

The data are available from the corresponding author upon reasonable request.
